# Byways of Medical Relief
*Previous articles in this series appeared in The Hospital of Oct. 7, Oct. 21, Nov. 18, and Dec. 2.


**Published:** 1912-01-06

**Authors:** 


### BYWAYS OF MEDICAL RELIEF.*

(Contributions are invited to this column.)

### V.—Little Homes for Ailing Children.

No form of charitable undertaking is more popular, none is more productive of permanent benefit, none is more simple to start and carry on, than the little home for children. Any dweller in the country with £100 or £200 to spare may build up a valuable centre of far-reaching benefit by hiring a cottage and fitting it up for the reception of children whose condition calls for special care. The supply of such children is unhappily overwhelming. As towns grow bigger the town life tends to become less and less adapted for rearing healthy children; or perhaps it would be better to say that knowledge of the measures needed to ensure a healthy childhood in crowded centres is advancing at a slower rate than the town population. A distant glimmering of perception begins to dawn that town life may be made less destructive to child development by the admission of more air and a healthier curriculum into the schools. But meantime the number of little victims to defective conditions increases, and it would seem that the only remedy open is to draft off those who are perceptibly flagging under them into pure air and country surroundings. The zealous co-operation of many who love little children has resulted in rescuing a countless number doomed apparently, without such aid, to premature death or disablement.

Little homes for children fall into two categories, preventive and curative. The preventive home requires the smallest possible amount of organisation, and can be run at very small cost. We have in mind a cottage in the grounds of a country villa. It is of irregular construction, built some four hundred years ago, and so oddly planned that it was marked out for destruction until it was bought and applied to its present purpose. Here, under the charge of a working matron, six little boys selected by the Jewish Board of Guardians in the East End are received all the year through. They come down for several months at a time until the weakness engendered by want or ill-health has completely disappeared. They attend the village school and lead a happy simple life.

All that is needed for establishing other such centres of recuperation is a healthily situated cottage, a kindly working matron in charge, good country provender, plenty of air and exercise, and, last but not least, care in the selection of the children. There must be some thoroughly experienced person to choose which children are to benefit, and the school master or mistress ought to be consulted to ensure that no degenerates are admitted among the others. There is very little risk of misconduct if proper care is taken, but it is the height of imprudence to retain children in small private homes of this description after it has once become evident that they are the victims of bad habits or are not amenable to mild discipline.

Curative homes for little children present more difficulties and are more costly to manage. Whether the cases selected be medical or surgical the presence of a trained nurse is required, and she will need a maid to do the housework and assist with the children, even in the smallest establishment. As in the case of chronic patients, much of the success of the treatment will depend on facilities for getting the little sufferers into the open air for the greater part of every day, and all the internal arrangements must be made to centre round this requirement. Wide verandahs or open-air shelters are indispensable for the hip and spinal cases, which are among the most pressing applicants for treatment. Cases of this nature demand treatment for long periods, and to effect a cure the child must sometimes remain two or three years. It thus becomes expedient to invite parents down to see their children, and maintain friendly intercourse with the relatives. Benevolent people are too apt to regard the natural guardians of their little charges with a jealousy which provokes hostility, forgetting that parents are directly responsible for the well-being of their offspring. The length of the course of treatment opens up also another source of difficulty. These children are unable to attend the ordinary village school, but it is a very serious matter if they are allowed to pass their time in idleness or in merely mechanical recreations. Their intellects are frequently rather above the normal, and they respond eagerly to instruction. If this is given by a voluntary worker, care should be taken to surround the periods of study with every formality. The strictest punctuality should be enforced, and there should be no intrusion of nursing detail into the school time. Even one hour a day screened off from every interruption counts for a great deal in the minds of these little sufferers. Some handicraft there should be also to occupy the fingers and perhaps prove subsequently a source of profit. All these matters demand a real sense of responsibility on the part of managers. It is not enough merely to place children in the best possible environment for recovery. There must be a reaching forward to the after-career when the treatment has succeeded and the once maimed child enters the ranks of ordinary workers.

Among the little homes which are doing fine work in rescuing the children fast drifting into the ranks of the incurable are the following: The Glynde Home for Convalescent Children at Lewes, with eight beds; the Charlton Cottage Home at Malmesbury, with eleven beds; the Sheet Cottage Home at Petersfield, Hants, with five beds; the Buttercups' Convalescent Home for Children at Twyford, with twelve beds; the Children's Surgical Home at Shanklin, with seven beds.

There should be no halting in the work of rescuing the little sufferers until in every town every elementary school is in touch with a cottage home to which ailing scholars could be despatched for restoration before the chronic stage sets in.

* Previous articles in this series appeared in The Hospital of Oct. 7, Oct. 21, Nov. 18, and Dec. 2.

